# Glycomics reveal that ST6GAL1‐mediated sialylation regulates uterine lumen closure during implantation

**DOI:** 10.1111/cpr.13169

**Published:** 2021-12-27

**Authors:** Kun Han, Feiyu Wang, Yulu Yue, Xihong Tan, Miao Tian, Yiliang Miao, Shuhong Zhao, Weijie Dong, Mei Yu

**Affiliations:** ^1^ Key Lab of Agricultural Animal Genetics, Breeding and Reproduction of Ministry of Education College of Animal Science and Technology Huazhong Agricultural University Wuhan China; ^2^ Institute of Stem Cell and Regenerative Biology College of Animal Science and Veterinary Medicine Huazhong Agricultural University Wuhan China; ^3^ College of Basic Medical Sciences Dalian Medical University Dalian China

**Keywords:** collective epithelial migration, N‐glycomic profile, pig, ST6GAL1, α2,6‐sialylation

## Abstract

**Objectives:**

Implantation failure is a major cause of prenatal mortality. The uterine lumen closure contributes to embryo adhesion to the uterus, but its underlying mechanisms are largely unknown. Our previous study has reported that endometrial fold extension can lead to uterine lumen closure in pigs. The objective of this study was to reveal molecular mechanisms of the uterine lumen closure by characterizing the molecular basis of the endometrial fold extension during implantation in pigs.

**Materials and methods:**

Uterine and endometrium tissues during implantation were collected in pigs. MALDI‐TOF MS was used to characterize the N‐glycomic profiles. Histochemistry, siRNA transfection, Western blotting, lectin immumoprecipitation, mass spectrometry and assays of wounding healing and cell aggregation were performed to investigate the molecular basis.

**Results:**

We observed that uterine luminal epithelium (LE) migrated collectively during endometrial fold extension. For the first time, we identified a large number of N‐glycan compositions from endometrium during implantation using MALDI‐TOF MS. Notably, the α2,6‐linked sialic acid and ST6GAL1 were highly expressed in uterine LE when the endometrial folds extended greatly. Subsequently, the role of ST6GAL1‐mediated 2,6‐sialylation in collective epithelial migration was demonstrated. Finally, we found that ST6GAL1‐mediated α2,6‐sialylation of E‐cadherin may participate in collective migration of uterine LE.

**Conclusions:**

The study reveals a mechanism of uterine lumen closure by identifying that ST6GAL1‐mediated α2,6‐sialylation of cell adhesion molecules contributes to endometrial fold extension through regulating collective migration of uterine LE.

## Graphical abstract

Effect of α2,6‐sialylation mediated by ST6GAL1 on regulation of pig endometrial folds extending during implantation. The ST6GAL1 mediated α2,6‐sialylation of cell adhesion molecular, such as E‐cadherin, alters the stability of the cell‐cell adherens junction, participates in activation of Rac1/WAVE1 signal pathway to regulate the collective migration of uterine LE and leads to the extending of endometrial folds during implantation.

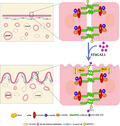

## 1 INTRODUCTION

Prenatal mortality caused by implantation failure is a common reproductive health concern for both humans and farm animals. It is of importance to elucidate the mechanisms of the implantation. During early implantation, the uterine lumen closure is a critical event contributing to embryo adhesion to the uterine LE. The morphological features and mechanisms of uterine lumen closure are well characterized in the mouse models,^1^, ^2^ but they remain unclear in humans and large animals due to the difficulty in collecting uterine samples during early implantation stage.

The litter‐bearing pig (*Sus scrofa*) is one of the most important farm animals. Besides, the pig serves as a large biomedical model to address various human health issues, including reproductive health.^3^ Pig blastocysts shed the zona pellucida around gestational days 6–7 (with entire gestational length of the pig of 114 days) and continue to develop into conceptuses (embryo/foetus and related extraembryonic membranes). The conceptuses freely float in the uterine lumen until gestational days 12–13 and then start to adhere to the uterine (LE) to form an epitheliochorial placenta around gestational day 18.^4^, ^5^ Previous studies have found that (1) when conceptuses remain free‐floating (before gestational day 12), the uterine lumen is in an open state and a few scattered endometrial folds occur; (2) at the stage of conceptus attachment (around gestational day 15), the endometrial folds that extend greatly to the uterine lumen interlock with each other, thus resulting in closure of the uterine lumen^5^, ^6^; (3) around gestational day 18, the uterine lumen reopens due to the expansion of chorioallantois.^4^ Therefore, the endometrial fold extension is, at least partially, the cause of the uterine lumen closure in pigs. Although a number of genes and pathways have been reported to be involved in implantation in pigs,^7^, ^8^ the mechanisms underlying the endometrial fold extension during implantation remain largely unknown.

Cell surface glycans participate in many important biological processes.^9^, ^10^, ^11^ The maternal‐foetal interface is enriched in various glycans,^12^, ^13^, ^14^ suggesting that glycans and glycan‐modified proteins play critical roles in embryo implantation. In pigs, some glycoproteins have been isolated and characterized during implantation.^15^, ^16^ By using lectin histochemistry approach, several glycans are found to be present in the pig maternal‐foetal interface throughout gestation.^17^, ^18^ However, the glycome (N‐ and O‐glycome) of the maternal‐foetal interface during implantation and the functions of glycans and glycan‐modified proteins in implantation remain to be investigated in large animals and humans.

The objective of this study was to reveal the molecular basis of the endometrial fold extension during implantation in pigs by investigating the N‐glycomic profiles of pig endometrium during implantation and characterizing the role of glycans in regulating the collective migration of uterine LE during implantation.

## 2 MATERIALS AND METHODS

### 2.1 Ethics statement

All animal procedures were approved by the Ethics Committee of Huazhong Agricultural University (HZAUSW‐2016–015).

### 2.2 Sample collection

Sample collection was carried out according to Wang et al.^6^ Briefly, Yorkshire gilts were mated to Yorkshire boars at the onset of the second estrus (Day 0) and again 12 h later. On gestational days 12, 15, and 18, the uterus was taken from each gilt and cut into segments (3–4 gilts/gestational day). Some of the uterine segments were randomly selected to be flushed with precooled RNase‐free phosphate buffer saline (PBS). When pregnancy was confirmed by the presence of conceptuses in the uterine flushing, the uterine segments in which the conceptuses were flushed out, were used for endometrium collection. The uterine segments that were not flushed were fixed immediately in 10% neutral‐buffered formalin followed by paraffin embedding.

### 2.3 Histological and histochemistry

The paraffin‐embedded intact uterine samples in which the conceptuses were not flushed out were cross‐sectioned into 4 μm thick using a Leica microtome (RM2235, Leica, Germany) and then deparaffinized and rehydrated by passage through xylene and a grade alcohol. Sections that were stained with haematoxylin and eosin were used for histological analysis.

Immunofluorescence assays were carried out as described by Wang et al.^6^ The primary antibodies used were ST6GAL1 (1:50, ab77676, abcam), ST6GAL2 (1:50, AF7747; R&D Systems), E‐cadherin (1:50, ab40772; abcam), β‐catenin (1:50, ab6302; abcam), Vimentin (1:50; sc‐73258), Rac1 (1:30, 66122–1‐Ig; Proteintech) and RhoA (1:50, 10749–1‐Ig; Proteintech). The fluorescent‐dye conjugated secondary antibodies used were Alexa Fluor^®^ 488 (1:100, ab150077/ab150113; abcam) or Alexa Fluor^®^ 555 (1:100, A21422/A21428; invitrogen). All slides were scanned by a Pannoramic Midi slide scanner (3D HISTECH; Budapest), and the image analysis was carried out by using CaseViewer 2.0 software (3D HISTECH).

### 2.4 Isolation of N‐linked glycans from pig endometrium

The endometrial tissues obtained from 3 gilts at each gestational day were pooled in equal weight (one pool/gestational day). Each pooled tissue sample (500 mg) was homogenized in denaturing buffer (0.5 M Tris‐HCl, pH 8.5, containing 7 M guanidine‐HCl and 10 mM EDTA) using an Ultrasonic Processor (Sonics VCX130, Sonics & Materials Inc). The sonicated solution was then centrifuged to collect the supernatant which contains proteins. The proteins (300 μg per sample) were reduced with 10 mM DL‐Dithiothreitol (DTT) at 60°C for 30 min, alkylated with 20 mM iodoacetamide (IAA) at room temperature for 60 min in the dark, dialysed against 10 mM ammonium bicarbonate (PH 8.6) for 48 h and digested with TPCK treated trypsin (T1426; Sigma) at 37°C for 18 h. After inactivation of trypsin by heat treatment (95°C for 5 min), 3 mU peptide N‐glycanase F (PNGase F, P0704S; New England Biolabs) was added to release the intact N‐glycans. The released N‐glycans were treated using the BlotGlyco^®^ glycan purification kit according to the manufacturer's protocol (Sumitomo Bakelite Co.). Briefly, the released N‐glycans were captured using BlotGlyco beads, followed by methylesterification of sialic acids with 3‐methyl‐1‐p‐tolytriazene (98%; Sigma‐Aldrich). Then, the captured N‐glycans were labelled and released with an aminooxy‐functionalized peptide reagent (aoWR). The derivatized glycans were recovered from the resin by washing with 50 μl of distilled water. Finally, excess reagent was removed using a cleanup column provided with the kit. The obtained solution containing the N‐glycan derivatives was analysed via mass spectrometry (MS).

### 2.5 Characterization of N‐glycomic profiles using MALDI TOF/TOF mass spectrometry

Mass spectra were acquired with a MALDI TOF/TOF mass spectrometer (New ultrafleXtreme; Bruker Daltonik). For sample preparation, 2,5‐Dihydroxybenzoic acid (DHB, 10 mg/ml in 30% ethanol) was used as the matrix and the DHB matrix (0.5 μl) was spotted onto a target plate (MTP 384 target plate ground steel; Bruker Daltonik) and dried. Subsequently, an aliquot (0.5 μl) of the N‐glycan solution was spotted onto the 2,5‐dihydroxybenzoic acid crystal and dried. Ions were generated by signal averaging over 4,000 laser shots from a Smartbeam‐II Nd:YAG laser operating at 355 nm and a repetition rate of 2 kHz. All spectra were obtained using the reflectron mode with a delayed extraction of 200 ns. The area of the isotopic peak of each glycan was normalized to an internal standard (maltoheptaose) with a known concentration. The ratio of each glycan's peak area to the sum of all glycans' peak area represents the content of a certain glycan. The glycan compositions were determined by database searching using GlycoWorkbench software.^19^ Briefly, exact mass of ‘labelling reagent’ was 432.22, and glycans labelled with the reagent were mainly detected as protonated molecular ion [M+H]^+^. Each of sialic acid residues was methyl‐esterified (mass +14.02). As a consequence, exact mass of glycans was calculated as glycan exact mass [M] = [observed m/z] – 432.22 + 18.01 – (14.02 × N) – 1.00 (N = number of sialic acid).

### 2.6 Lectin‐histochemical analysis

The lectin‐histochemical study was performed on pig uterine cross sections. Slides were incubated with either fluorescein isothiocyanate (FITC) labelled lectin *Sambucus nigra* (SNA, 1:150, FL‐1391; Vector Labs) or biotin‐conjugated lectin *Maackia amurensis* (MAL‐II, 1:150, B‐1265; Vector Labs) at 4°C overnight. The biotin‐conjugated lectin was subsequently detected with streptavidin‐FITC conjugate (1:100, SA‐5001; Vector Labs). Nuclei were stained with DAPI (G1012; Servicebio) and sealed with anti‐fluorescence quenching tablets. All slides were scanned by a Pannoramic Midi slide scanner (3D HISTECH), and the image analysis was carried out by using CaseViewer 2.0 software (3D HISTECH).

### 2.7 Cytochemistry analysis

Ishikawa cells were cultured in DMEM (C11995500BT; Gibco) with 10% foetal bovine serum (FBS, SE200‐ES; VisTech™) and 1% antibiotics (15240062; Gibco). Cells were seeded at a density of 1 × 10^5^ cells on 12‐well glass bottom plates and fixed with 4% paraformaldehyde for 10 min, washed three times with cold PBS and permeabilized with 0.1% Triton X‐100 in PBS for 10 min. After being washed with cold PBS for 3 times, cells were blocked with PBST buffer containing 1% BSA and 10% goat serum for 2 h at room temperature.

For immunofluorescence assays, cells were incubated with primary antibodies at 4 °C overnight. The primary antibodies used were ST6GAL1 (1:50; ab77676, abcam), E‐cadherin (1:50; ab40772, abcam), β‐catenin (1:50; ab6302, abcam), Vimentin (1:50; sc‐73258), Rac1 (1:30, 66122–1‐Ig; Proteintech) and RhoA (1:50, 10749–1‐Ig; Proteintech). Then, cells were incubated with the fluorescent‐dye conjugated secondary antibodies (Alexa Fluor^®^ 488, ab150077/ab150113; abcam or Alexa Fluor^®^ 555, A21422/A21428; invitrogen) at 1:100 dilutions in PBST containing 1% BSA.

For lectin staining, cells were incubated with FITC labelled SNA (1:150, FL‐1391; Vector Labs) or biotin‐conjugated MAL‐II (1:150, B‐1265; Vector Labs). The biotin‐conjugated MAL‐II was detected with streptavidin‐FITC conjugate (1:100, SA‐5001; Vector Labs). Imaging of cells was completed using the fluorescent microscope (BX53; Olympus Corporation).

### 2.8 Lectin inhibition assay and siRNA transfection

To investigate the effect of α2,6‐sialylation on cell migration and cell‐cell adhesion, two approaches were used: (1) treatment of cells with SNA (1:100, L‐1300; Vector Labs), a lectin that can specifically bind to α2,6‐linked sialic acid residues, to block the role of α2,6‐linked sialic acids. (2) siRNA‐mediated knockdown of *ST6GAL1* to decrease the levels of α2,6‐linked sialic acids. *ST6GAL1* siRNA (si‐*ST6GAL1*) oligonucleotides (sense: 5′‐CAGCCAACUUCCAACAAGAdTdT‐3′/antisense: 5′‐dTdTGUCGGUUGAAGGUUGUUCU‐3′) and non‐targeting control siRNA (si‐NC) oligonucleotides (sense: 5′‐UUCUCCGAACGUGUCACGUTT‐3′/ antisense: 5′‐ACGUGACACGUUCGGAGAATT‐3′) were synthesized by GenePharma. Briefly, Ishikawa cells were transfected with a final concentration of 110 pmol (for a 6‐well plate) or 55 pmol (for a 12‐well plate) of si‐*ST6GAL1* and si‐NC oligonucleotides using jetPRIME siRNA transfection reagent (114–15, Polyplus‐transfection^®^ SA) according to the manufacturer's instructions. Quantitative RT‐PCR (qRT‐PCR) and Western blot were performed after cells were transfected with si‐*ST6GAL1* or si‐NC for 72 h.

### 2.9 qRT‐PCR

Total RNA was isolated from pig endometrium and Ishikawa cells using RNeasy Plus Mini Kit (74134; Qiagen) following the instructions provided by the manufacture. The cDNA was synthesized using PrimeScript RT Reagent Kit with gDNA Eraser (RR047B; Takara Biomedical Technology). qRT‐PCR was performed with Bio‐Rad CFX96 real‐time PCR system using SYBR Premix Ex Taq (RR420A; Takara Biomedical Technology). GAPDH was used as the internal reference. All primer sequences are shown in Table S1.

### 2.10 Western blotting

Protein extraction from tissues or cells was performed using RIPA lysis buffer (P0013D; Beyotime) containing 0.1% protease inhibitor PMSF (100mM, ST506; Beyotime). The proteins were separated in SDS‐PAGE gel and transferred onto PVDF membranes (0.22 µm Millipore, ISEQ00010; biosharp). The non‐specific binding sites on the membranes were blocked with 5% skim milk in TBS‐Tween 0.1% for 3 h at room temperature. Membranes were then incubated with primary antibodies diluted in antibody diluent or biotin‐conjugated SNA (1:2500, B‐1305, Vector Labs;) at 4°C overnight. Then, membranes were washed with TBST 5 times for 10 min each and incubated with horseradish peroxidase (HRP) conjugated goat anti‐rabbit (1:2,000, A0208; Beyotime) or anti‐mouse secondary antibody (1:2,000, A0216; Beyotime) or streptavidin (1:2,000, SA00001‐0; Benchmark) or Clean‐Blot IP Detection Reagent (HRP) (1:500, 21,230; Thermo Scientific) for 2 h at room temperature. After membranes were washed with TBST 5 times for 10 min each, proteins were detected using the ECL Western blot kit (170–5,060; Bio‐red) and analysed by a chemiluminescent imaging system (Tanon‐5200; Tanon Science and Technology). β‐actin or GAPDH was used as the internal reference. The primary antibodies used included ST6GAL1 (1:2500; ab77676, abcam), E‐cadherin (1:2,500; ab40772, abcam), Rac1 (1:2,500, A7720; abclonal), WAVE1 (1:2,500; ab185546, abcam), β‐actin (1:2500, AF5003; Beyotime) and GAPDH (1:2500, AF1186; Beyotime).

### 2.11 Wounding healing assay

Ishikawa cells were seeded in a 6‐well plate (6 × 10^5^) and maintained in DMEM containing 2% FBS and 1% antibiotics for 12–16 h. After starvation, confluent cell monolayers were wounded with a sterile 10 µl pipette tip and photographed immediately under a microscope (BX53; Olympus Corporation). Then, the cells were either treated with SNA or transfected with si‐*ST6GAL1* or si‐NC. After 24 h, 48 h and 72 h, images were taken using the microscope (BX53; Olympus Corporation). Three independent experiments were performed. The wound‐healing area was analysed using Image J software (National Institutes of Health, Bethesda, MD). The relative healing area percentage was calculated following the formula: the relative cell migration % = (the wound area at 0 h‐the wound area at 24, 48 or 72 h)/the wound area at 0 h × 100%.^20^

### 2.12 Cell aggregation assay

Semi‐solid agar medium was prepared by dissolving 100 mg agar (121985; Gene Company) into 15 ml Ringer's salt solution (SL64381; Coolaber science & technology). An aliquot of 50 µl semi‐solid agar medium was transferred into each well of a 96‐well plate, and the plate was placed at 4°C to let them solidify. Ishikawa cells were washed twice with Moscona solution, digested by trypsin and seeded at the density of 6 × 10^4^ on a 96‐well plate pre‐coated with solidified agar. This plate was placed in a cell incubator (37°C in a humidified atmosphere with 5% CO_2_ in air) for 24 h. The aggregation was evaluated under a microscope at 4× magnification (BX53; Olympus Corporation). The cells were counted from three fields, and three independent experiments were performed.

### 2.13 Lectin immumoprecipitation

A total of 500 mg pig endometrium from gestational days 12, 15 and 18 or 1 × 10^7^ Ishikawa cells were lysed in 500 µl RIPA lysis buffer (P0013D; Beyotime) containing 0.1% protease inhibitor PMSF (100 mM, ST506; Beyotime) and centrifuged at 12879 *g* for 30 min at 4°C to obtain the supernatant. Then, a total of 350 µl of supernatant were incubated with SNA‐conjugated agarose beads (200 µl, AL‐1303; Vector Labs) at 4°C overnight to obtain the immunoprecipitated proteins. After being washed three times with RIPA lysis buffer, the immunoprecipitated proteins were subjected to Western blot and liquid chromatography with tandem mass spectrometry mass spectrometry (LC‐MS/MS). For Western blot, three endometrial samples from each gestational day (n = 3 gilts/gestational day) and three independent cell samples were used. Briefly, the immunoprecipitated proteins were separated by SDS‐PAGE on a 10% gel, transferred to PVDF membranes (0.22 µm Millipore, ISEQ00010; biosharp) and then the membranes were probed with anti‐E‐cadherin. The immunoprecipitated proteins that were probed with SNA was used as the loading control. In addition, the expression level of E‐cadherin in proteins that were not immunoprecipitated were detected in parallel and β‐actin (1:2500, AF5003; Beyotime) was used as the loading control. Proteins were detected using the ECL Western blot kit (170–5,060; Bio‐red) and analysed by a chemiluminescent imaging system (Tanon‐5200; Tanon Science and Technology).

### 2.14 Identification of α2,6‐sialylated proteins using mass spectrometry analysis

The proteins immunoprecipitated from pig endometrium of gestational day 15 (n = 3 gilts) were subjected to LC‐MS/MS analysis in PTM‐BIO Company. Briefly, the immunoprecipitated proteins were separated by SDS‐PAGE and stained by coomassie blue. After cutting, the gel was treated with DTT, IAA and trypsin to obtain peptides. The peptides were desalted by C18 tip and detected by Easy‐nLC 1000‐LTQ Orbitrap ETD MS/MS (Thermo Fisher, Bremen, Germany) analysis. The resulting mass spectrometer data were searched against the UniProt *Sus Scrofa* database (https://www.uniprot.org/) using the Proteome Discoverer 1.4 software. Data from the three independent endometrial samples were combined and subjected to gene ontology (GO) analysis using g:Profiler (http://baderlab.org/Software/EnrichmentMap/GProfilerTutorial).

### 2.15 Statistical analysis

Statistical analyses were carried out using GraphPad Prism Software version 5 (GraphPad Software). The statistical significance of differences was analysed using nonparametric Mann‐Whitney one tail/two tail test. The data were expressed as means ±standard error. *p* < 0.05 was considered statistically significant. All graphs were generated using GraphPad Prism software.

## 3 RESULTS

### 3.1 Uterine LE migrates collectively during endometrial fold extension

**FIGURE 1:**
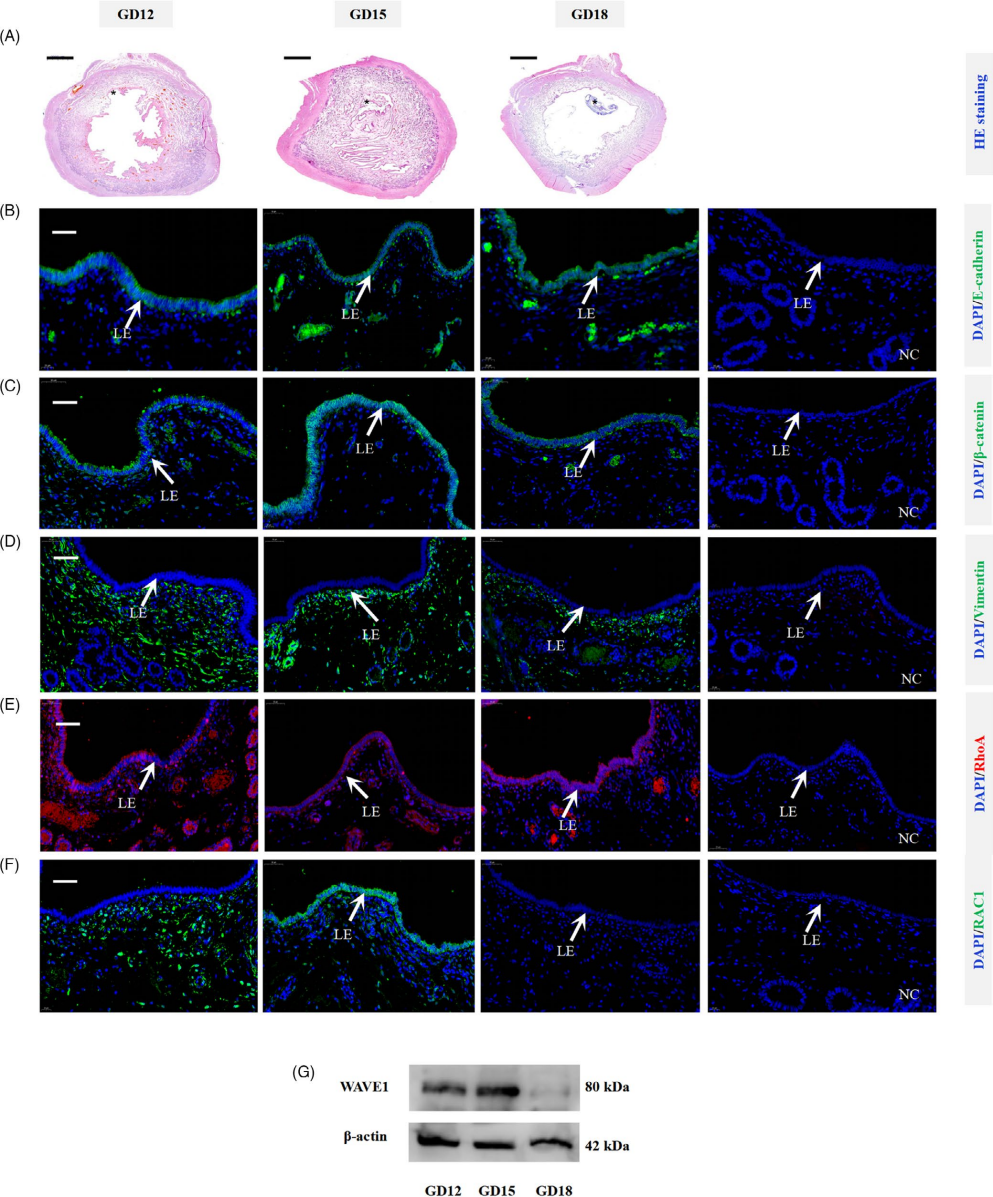
Expression patterns of the collective epithelial migration markers in uterine LE during implantation in pigs. (A) Representative images of haematoxylin and eosin‐stained pig uterine cross sections from gestational days 12, 15 and 18 (n = 3 gilts/gestational day). On gestational day 12, the uterine lumen is in an open state. On gestational day 15, the endometrial folds extend greatly towards uterine cavity and the uterine lumen is in a closure state. On gestational day 18, the uterine lumen reopens. The embryo is marked with an asterisk (*). Scale bar = 2,000 µm. HE, haematoxylin and eosin. (B‐F) Expression patterns of E‐cadherin, β‐catenin, vimentin, Rac1 and RhoA in uterine LE during implantation in pigs (n = 3 gilts/ gestational day). The positive signal is in green (E‐cadherin, β‐catenin, vimentin, and Rac1) or rose red (RhoA), while the nucleus is in blue. Scale bar = 50 µm. NC, negative control. (G) Representative Western blots showing the expression of WAVE1 in pig endometrium from gestational days 12, 15 and 18 (n = 3 gilts/ gestational day). β‐actin was used as the loading control. GD, gestational day. LE, luminal epithelium

The histological analysis of pig uterine cross section revealed that the uterine lumen experienced a process of ‘open‐close‐reopen’ on gestational days 12, 15 and 18 (Figure 1A). Then, a total of six cell migration markers were used to mark the epithelial to mesenchymal transition (E‐cadherin, β‐catenin and vimentin) and cell migration (RhoA, Rac1 and WAVE1).^21^, ^22^, ^23^, ^24^ The immunofluorescence assays revealed that (1) E‐cadherin and β‐catenin were consistently expressed at the cell‐cell boundaries of uterine LE (Figure 1B, C), while vimentin was observed in stroma but not in uterine LE on gestational days 12, 15 and 18 (Figure 1D). (2) RhoA was expressed in uterine LE at these 3 days (Figure 1E), and Rac1 was expressed in stratum compactum stroma on gestational day 12 and only expressed in uterine LE on gestational day 15 (Figure 1F). In addition, Western blotting analysis revealed that WAVE1 was expressed in pig endometrium on gestational days 12 and 15 (Figure 1G). Taken together, these results showed that E‐cadherin, β‐catenin and RhoA were consistently expressed in uterine LE, while Rac1 and WAVE1 were expressed on gestational day 15 in uterine LE and endometrium, respectively, indicating that the uterine LE might migrate collectively at which the endometrial folds extend greatly.

### 3.2 Sialylated glycans are highly expressed in endometrium on gestational days 12, 15 and 18 by MALDI‐TOF MS

**FIGURE 2:**
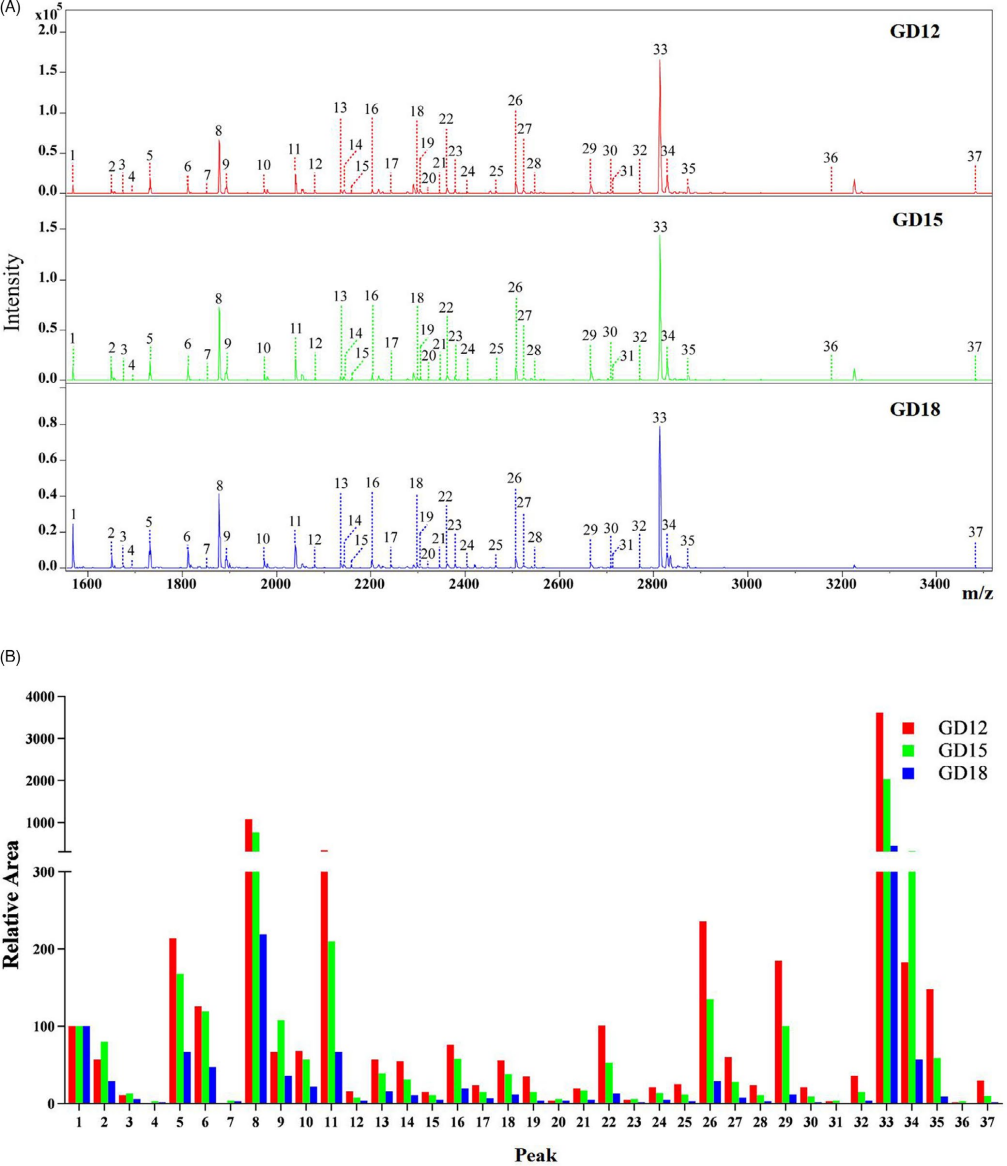
MALDI‐TOF MS analysis of N‐glycans released from pig endometrium during implantation. (A) MALDI‐TOF MS spectra of N‐glycans released from pig endometrium on gestational days 12, 15 and 18. The Y axis depicts the signal intensity of N‐glycan in arbitrary units as determined by MS spectra. The peaks marked with the arabic numerals were assigned to the corresponding glycan compositions and summarized in Table S1. (B) Histogram of the relative area of each peak. The area of peak 1, which is the internal standard (maltoheptaose) with a known concentration, was defined as 100. GD, gestational day

The N‐glycan profiles of pig endometrium on gestational days 12, 15 and 18 were characterized by MALDI‐TOF MS respectively (Figure 2A). Each peak in the MS spectra was assigned to the corresponding glycan composition (Table S2). To simplify the comparison of the profiles of the released glycans on gestational days 12, 15 and 18, the area of the isotopic peaks of each glycan (peak 2–37) in the MS spectra was normalized to an internal standard with a known concentration (peak 1) and represented as a histogram (Figure 2B, Table S2). A total of 36 possible N‐glycan compositions were estimated, including 5 high mannose‐type (peak 2, 6, 10, 13 and 18), 8 hybrid‐type (peak 3, 4, 7, 14, 15, 19, 20 and 25) and 23 complex‐type (Table S2). These 23 complex‐type glycans consist of 13 bi‐antennary (peak 5, 8, 9, 11, 16, 21, 22, 23, 26, 27, 29, 33 and 34), 6 tri‐antennary (peak 17, 28, 31, 35, 36 and 37), 1 tetra‐antennary (peak 32) and 3 bisecting (peak 12, 24 and 30) complex‐type N‐glycans. Thus, the identified N‐glycans cover all the 3 types of N‐glycan. In addition, the most intense glycan signal at every gestational day is peak 33, which has two Neu5Ac residues and one fucose residue. The content of the glycan was 50.7%, 43.3% and 34.4% on gestational days 12, 15 and 18 respectively. Additionally, the number of sialylated glycans on each of these 3 days was more than half of all the detected glycans, especially after peak 19 (except peak 24 and 32).

### 3.3 α2,6‐linked sialic acid glycan and ST6GAL1 are highly expressed in uterine LE during endometrial fold extension

**FIGURE 3:**
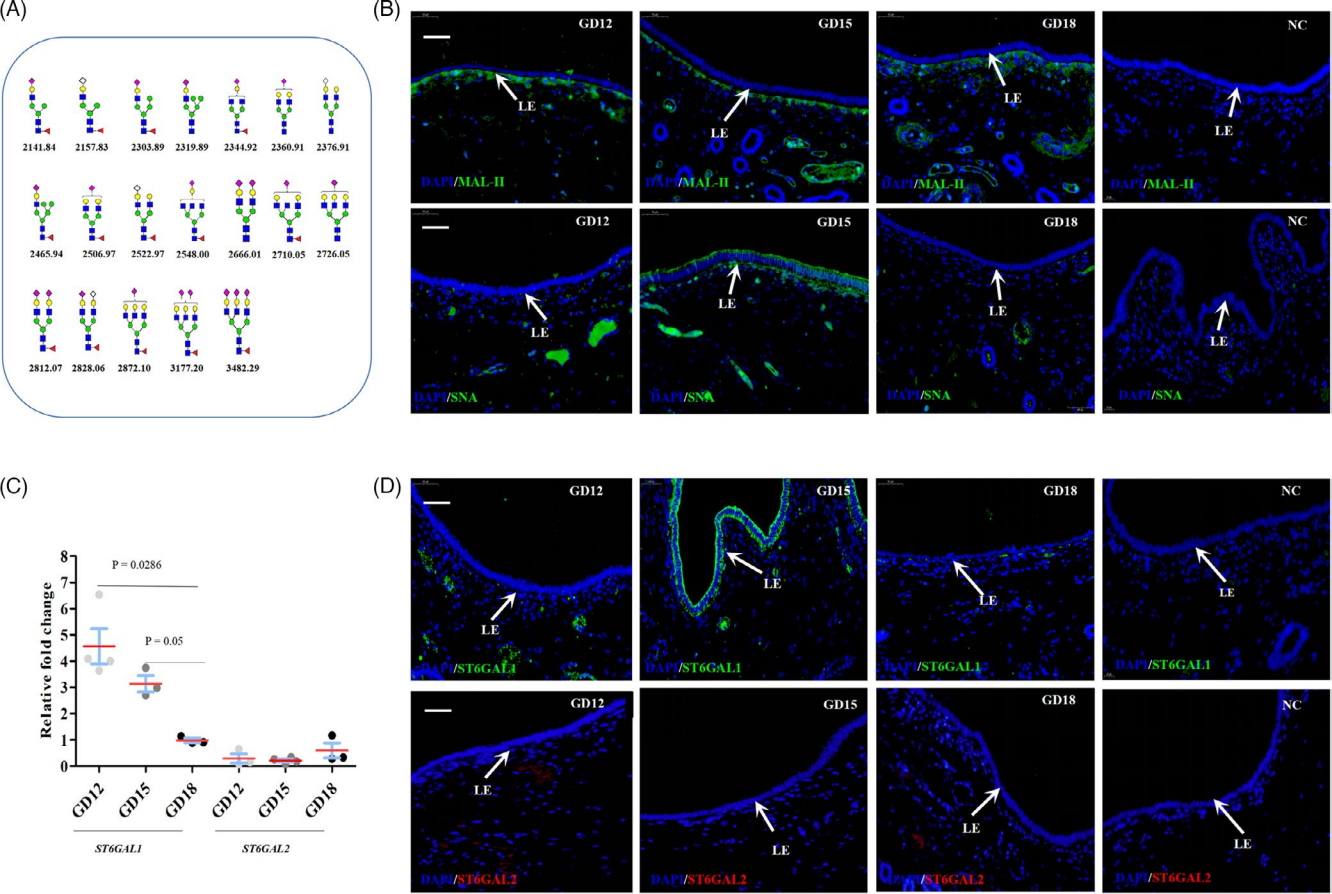
Expression patterns of the sialylated glycans as well as the mRNA and protein expression of ST6GAL1 and ST6GAL2 in pig endometrium during implantation. (A) The 19 possible α2,3/6‐sialylated N‐glycans identified by MALDI‐TOF MS. Fucose 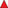; N‐acetylglucosamine 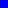; N‐acetyl neuraminic acid 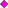; N‐glycolyl neuraminic acid 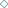; mannose 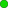; Galactose 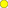. (B) Representative images of lectin fluorescence assays taken from pig endometrium on gestational days 12, 15 and 18 (n = 3 gilts/gestational day). MAL‐II was used to detect α2,3‐linked sialic acid residues. SNA was used to detect α2,6‐linked sialic acid residues. (C) Expression levels of *ST6GAL1* and *ST6GAL2* in pig endometrium on gestational days 12, 15 and 18 measured by qRT‐PCR (n = 3 gilts/gestational day). The error bars represent the standard error. Mean is denoted by a red line. Nonparametric Mann‐Whitney one tail test was used for statistical analysis. (D) Distributions of ST6GAL1 and ST6GAL2 in pig endometrium on gestational days 12, 15 and 18 (n = 3 gilts/gestational day). The positive signal is in green, while the nucleus is in blue. NC, negative control. LE, endometrial luminal epithelium. GD, gestational day. Scale bar = 50 µm

As shown in Figure 3A, a total of 19 possible N‐glycan compositions detected by MALDI‐TOF MS were identified as the α2,3/6‐sialylated N‐glycans. The linkage types and distributions of the sialylated glycans were determined by lectin fluorescence assay in pig uterine cross sections. Lectins *Maackia amurensis* (MAL‐II) and *Sambucus nigra* (SNA) were used to detect α2,3‐ and α2,6‐linked sialic acid residues respectively.^25^ The MAL‐II stain signal was observed in subepithelial fibroblasts, stroma and blood vessel walls, but not in uterine LE and glandular epithelium (Figure 3B, Figure S1). The SNA stain signal was detected in glandular lumen and blood vessel. In uterine LE, SNA stain signal was weak on gestational day 12 and strong on day 15, but undetectable on day 18 (Figure 3B, Figure S1). Collectively, the α2,6‐linked sialic acid glycan is expressed in uterine LE in a stage‐specific manner during implantation.

Further, we investigated the expression pattern of two β‐galactoside α2,6‐sialyltransferases, ST6GALI and ST6GALII, which are responsible for transferring sialic acid to Galβ1,4GlcNAc (N‐acetyllactosamine) through α2,6‐linkage.^26^ The qRT‐PCR results showed that the expression level of *ST6GAL1* in pig endometrium was significantly higher on gestational days 12 and 15 than on day 18 (Figure 3C, two‐sample nonparametric Mann‐Whitney one tail test). Further immunofluorescence assay revealed that the expression level of ST6GAL1 in uterine LE was low on day 12 and was high on day 15, but it was undetectable on day 18 (Figure 3D). In addition, ST6GAL1 was detectable in glandular lumen and blood vessel during these 3 gestational days (Figure S2). In contrast to ST6GAL1, neither ST6GAL2 mRNA nor ST6GAL2 protein was detectable in pig endometrium during these 3 gestational days (Figure 3C, 3D and Figure S2). These results suggest that the expression of α2,6‐linked sialic acid in pig endometrium is mediated by ST6GAL1. In addition, the expression pattern of α2,6‐linked sialic acid was consistent with that of ST6GAL1 in uterine LE, and their expression pattern was stage‐specific.

### 3.4 α2,6‐sialylation promotes cell migration and cell‐cell adhesion

**FIGURE 4:**
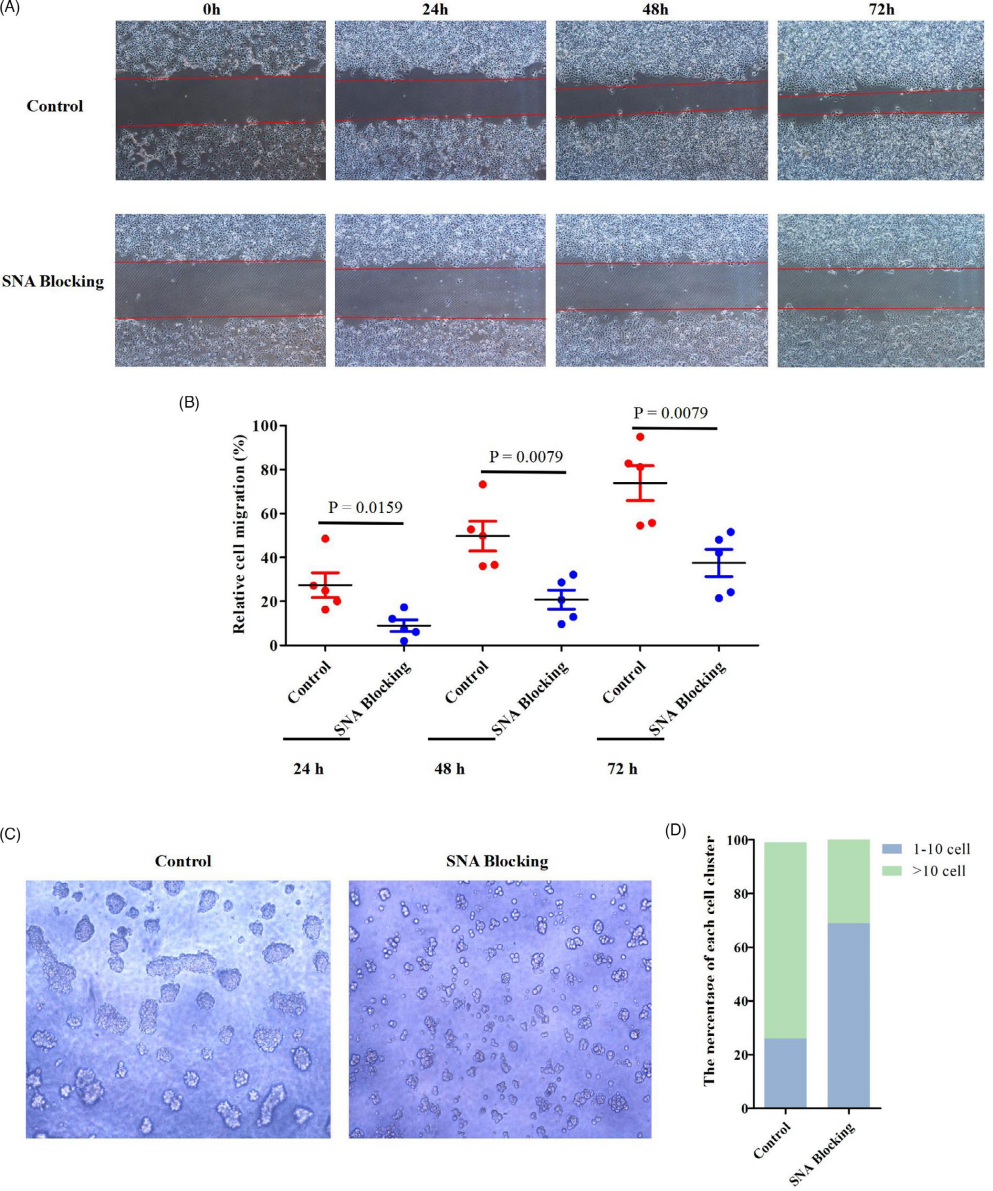
Effect of α2,6‐sialylation on cell migration and cell adhesion. Representative images of wound‐healing assays in Ishikawa cells treated with SNA for 0, 24, 48 and 72 h (4×magnification). (B) Relative cell migration of the SNA treated Ishikawa cells. The error bars represent the standard error. Mean is denoted by a black line. Nonparametric Mann‐Whitney test was used for statistical analysis (n = 3 independent experiments). (C) Representative bright‐field images of aggregates (100× magnification). (D) Quantification of cell aggregates. Cells were binned into cluster classes: 1–10 cells and >10 cells. The percentage of cells in each cluster size for each group is indicated (n = 3 independent experiments)

Fluorescent staining with SNA and MAL‐II revealed that α2,6‐linked sialic acid rather than α2,3‐linked sialic acid was expressed in Ishikawa cells. In addition, staining signals of ST6GAL1 and E‐cadherin were also observed in Ishikawa cells (Figure S3). To investigate the effect of α2,6‐sialylation on cell migration and cell‐cell adhesion, the α2,6‐linked sialic acids were blocked through cellular SNA treatment. As shown in Figure S4, the α2,6‐linked sialic acid expression levels were decreased in Ishikawa cells after SNA treatment. Wound‐healing assay results indicated that blocking of cellular α2,6‐linked sialic acids by SNA treatment resulted in a decrease in the ability of wound healing at 24 h, 48 h and 72 h post‐treatment (Figure 4A, B, two‐sample nonparametric Mann‐Whitney test). In addition, cell aggregation assay showed that only ~26% of the aggregates containing more than 10 cells formed in the cells whose α2,6‐linked sialic acids were blocked, but ~69% of the aggregates formed in Ishikawa cells whose α2,6‐linked sialic acids were not blocked (Figure 4C, D). The results suggest that the presence of α2,6‐sialylation promotes cell migration and cell‐cell adhesion.

### 3.5 ST6GAL1‐mediated α2,6‐sialylation of E‐cadherin contributes to collective cell migration

**FIGURE 5:**
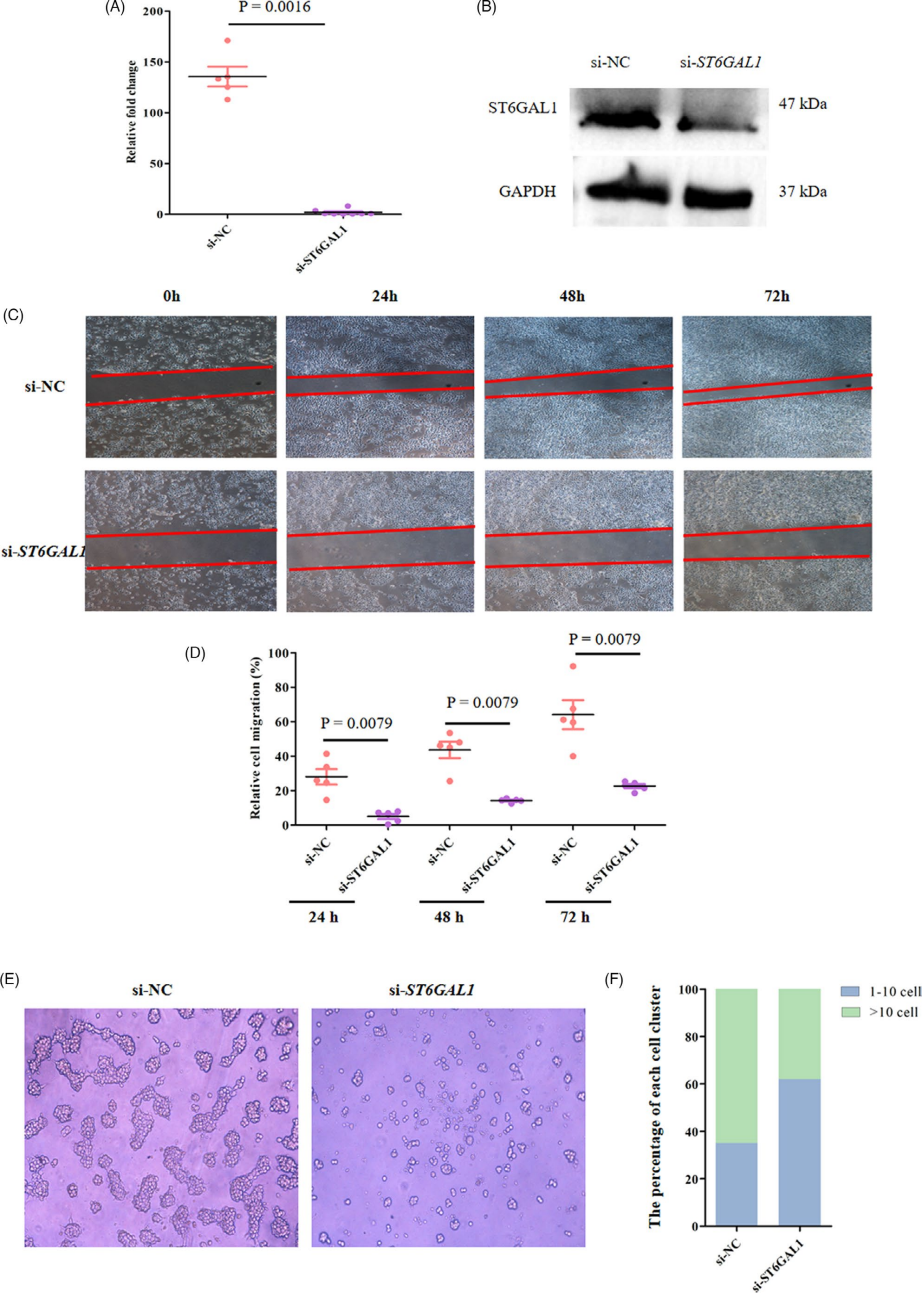
Effect of ST6GAL1 on cell migration and cell adhesion. The mRNA expression of *ST6GAL1* in Ishikawa cells transfected with si‐*ST6GAL1* or si‐NC. The error bars represent the standard error. Mean is denoted by a black line. Nonparametric Mann‐Whitney test was used for statistical analysis. (B) Representative Western blots showing the expression of ST6GAL1 in Ishikawa cells transfected with si‐*ST6GAL1* or si‐NC. (C) Representative images of wound‐healing assays in Ishikawa cells transfected with si‐*ST6GAL1* or si‐NC for 0, 24, 48 and 72 h (4× magnification). (D) Relative cell migration of Ishikawa cells transfected with si‐*ST6GAL1* or si‐NC. The error bars represent the standard error. Mean is denoted by a black line. Nonparametric Mann‐Whitney test was used for statistical analysis. (E) Representative bright‐field images of aggregates formed in Ishikawa cell transfected with si‐*ST6GAL1* or si‐NC for 24 h (100× magnification). (F) Quantification of cell aggregation assays. Cells were binned into cluster classes: 1–10 cells and >10 cells. The percentage of cells in each cluster size for each group is indicated. n = 3 independent experiments

**FIGURE 6:**
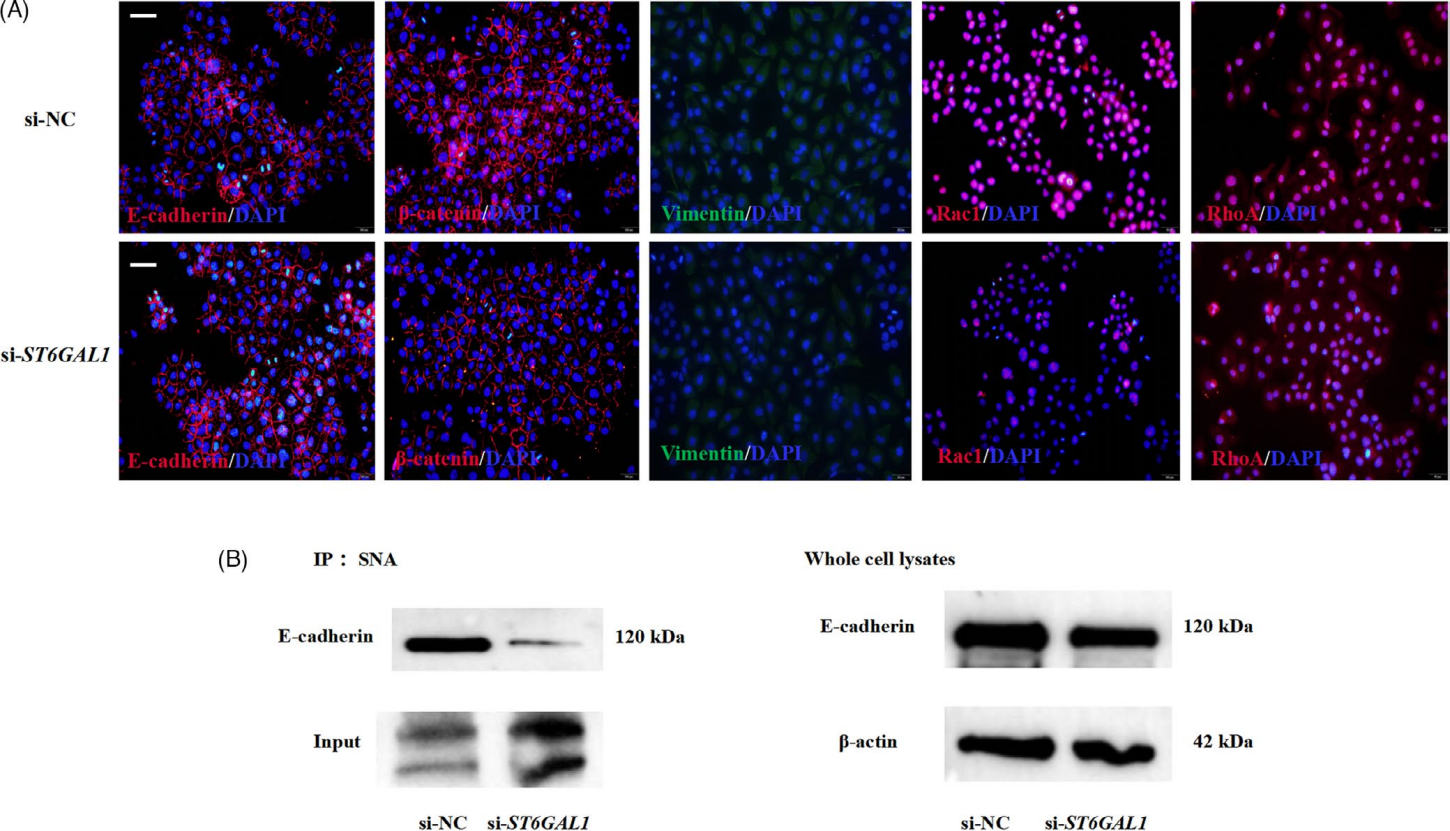
Effect of ST6GAL1‐mediated α2,6‐sialylation of E‐cadherin on collective cell migration. (A) Knockdown of ST6GAL1 affects the expression of collective migration marker proteins. Representative images showing expression of E‐cadherin, β‐catenin, vimentin, Rac1 and RhoA in Ishikawa cells transfected with si‐*ST6GAL1* or si‐NC for 72 h. The positive signal is in rose red, while the nucleus is in blue (n = 3 independent experiments. 20× magnification). (B) The α2,6‐sialylated proteins were pulled down with SNA lectin from Ishikawa cell lysates, and Western blotting was performed to evaluate the α2,6‐sialylation levels of E‐cadherin. The immunoprecipitated proteins that were probed with SNA were used as loading controls (left panel). The whole cell lysates were probed with an antibody against E‐cadherin to show the amounts of E‐cadherin, and β‐actin was used as a loading control (right panel). n = 3 independent experiments. IP, immunoprecipitation. WB, Western blot.

The small interfering RNA (siRNA) technique was used to knock down the expression of *ST6GAL1* at mRNA and protein levels in Ishikawa cells (Figure 5A, B). Fluorescent staining results showed that the cells transfected with si‐*ST6GAL1* showed a lower expression level of α2,6‐linked sialic acid and ST6GAL1 than those transfected with si‐NC, indicating the successful knockdown (Figure S5A, B). Wound‐healing assay showed that the knockdown of *ST6GAL1* by siRNA significantly decreased the ability of wound healing after 24‐h, 48‐h and 72‐h transfection (Figure 5C, D, two‐sample nonparametric Mann‐Whitney test). The cell aggregation assay indicated that only ~38% of the aggregates containing more than 10 cells formed in the cells transfected with si‐*ST6GAL1*, whereas ~65% of the aggregates formed in the cells transfected with si‐NC (Figure 5E, F). Immunofluorescence assays showed that E‐cadherin and β‐catenin were expressed at the cell‐cell boundaries in both si‐*ST6GAL1*‐transfected cells and si‐NC‐transfected cells. In contrast, vimentin was expressed in neither si‐*ST6GAL1*‐transfered cells nor si‐NC‐transfected cells (Figure 6A). However, the cells transfected with si‐*ST6GAL1* showed decreased level of Rac1, compared with the cells transfected with si‐NC (Figure 6A). As shown in Figure 6A and B, the level of E‐cadherin extracted from Ishikawa cells transfected with si‐*ST6GAL1* was consistent with that from Ishikawa cells transfected with si‐NC. However, the level of E‐cadherin in the SNA immunoprecipitates from the cells transfected with si‐*ST6GAL1* was lower than that from the cells transfected with si‐NC (Figure 6B). The above results indicated that the knockdown of *ST6GAL1* by siRNA resulted in decreased α2,6‐sialylation of E‐cadherin. Taken together, the above findings suggest that ST6GAL1‐mediated α2,6‐sialylation of E‐cadherin facilitates collective cell migration.

### 3.6 E‐cadherin is highly α2,6‐sialylated in uterine LE during the endometrial fold extension

**FIGURE 7:**
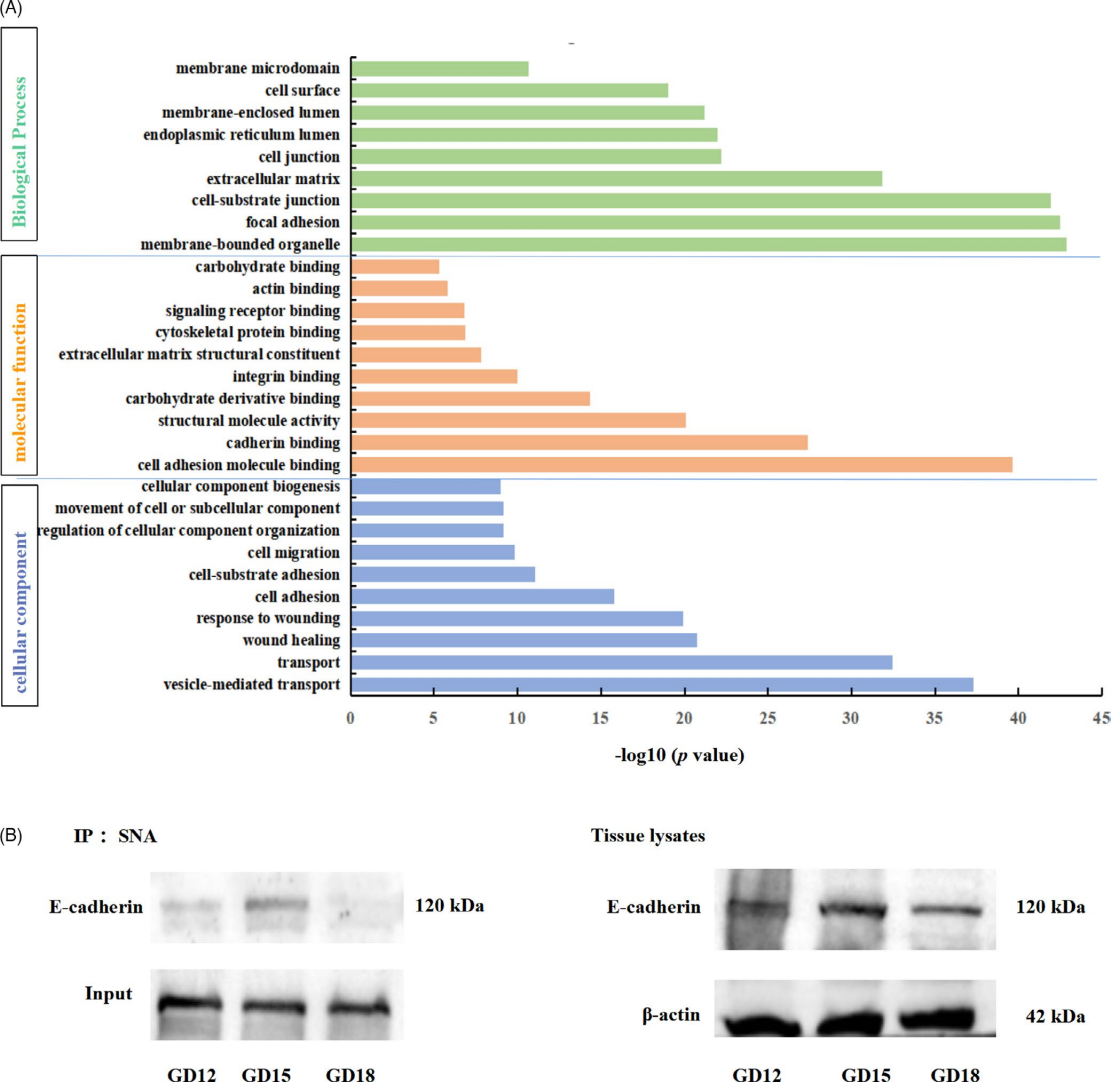
Identification of the changes in α2,6‐sialylation levels of proteins from pig endometrium. (A) GO analysis of the α2,6‐sialylated proteins identified from pig endometrium on gestational day 15. (B) The α2,6‐sialylated proteins from pig endometrium were pulled down with SNA lectin, and then, Western blotting was performed to evaluate the α2,6‐sialylation levels of E‐cadherin. The immunoprecipitated proteins that were probed with SNA were used as loading controls (left panel). Pig endometrium lysates were probed with an antibody against E‐cadherin to show the amounts of E‐cadherin, and β‐actin was used as a loading control (right panel). n = 3 gilts/gestational day. IP, immunoprecipitation. WB, Western blot. GD, gestational day

To identify the α2,6‐sialylated proteins in pig endometrium, the α2,6‐sialylated proteins were pulled down with SNA lectin from pig endometrium on gestational days 12, 15 and 18. The precipitated α2,6‐sialylated proteins from gestational day 15 were determined by LC‐MS/MS (n = 3 gilts). A total of 796 α2,6‐sialylated proteins were identified to be mainly enriched in functional terms related to cell adhesion (Figure 7A, Table S3). It is worth noting that E‐cadherin, one of the collective cell migration markers, is in the list of these identified α2,6‐sialylated proteins. We further examined the change in the α2,6‐sialylation level of E‐cadherin in pig endometrium on gestational days 12, 15 and 18. The Western blotting analyses showed that the expression level of E‐cadherin was similar in endometrium during these 3 gestational days, but it was much higher in SNA immunoprecipitates from endometrium lysates on gestational day 15, the day when the endometrial folds extend greatly (Figure 7B).

## 4 DISCUSSION

In pigs, endometrial fold extension, as a morphological change of endometrium, can lead to the uterine lumen closure during attachment time (around gestational day 15). Cell migration is crucial in tissue morphogenesis, and it can be classified into two types: individual and collective cell migration. The critical event that allows epithelial cells to acquire the ability of individual cell migration is the process of epithelial‐mesenchymal transition (EMT). The hallmark of collective cell migration is that the cells retain junctions with neighbour cells during movement.^27^, ^28^, ^29^ Thus, collective cell migration is a process in which a group of cells migrate cohesively as a multicellular unit. A number of studies have demonstrated that collective cell migration plays important roles in tissue morphogenesis and remodelling.^30^, ^31^, ^32^ Endometrium is a multicellular tissue composed of epithelial and stromal tissue compartments. In this study, we observed that E‐cadherin and β‐catenin were consistently expressed in pig uterine LE but vimentin was not, indicating that the uterine luminal epithelial cells retained cell‐cell junctions and did not transdifferentiate into mesenchymal cells during implantation. In addition, cell migration involves five steps: cell polarization, membrane extension, cell‐substratum adhesions, cell‐body translocation and rear retraction.^28^, ^29^ We further observed that RhoA was expressed in uterine LE on the 3 gestational days, whereas Rac1 was expressed in uterine LE only on gestational day 15, the day when the endometrial folds extended greatly to the uterine lumen and undetectable on gestational days 12 and 18 when uterine lumen was in an open state. Rac1 and RhoA are the two members of Rho GTPase family. Rac1 activates formation of lamellipodia and membrane ruffles, while RhoA controls actomyosin contraction and cell contractility.^33^, ^34^ Rac1 and RhoA work coordinately to regulate actin reorganization which is essential for cell migration through activation of WAVE1, a downstream effector for Rac.^35^, ^36^ As expected, WAVE1 was expressed in pig endometrium on gestational day 15, suggesting that WAVE1 was activated by active Rac1. Based on the observation that pig uterine LE maintains the cell‐cell connection but exhibits Rac1 pathway‐mediated migration, we conclude that the uterine luminal epithelial cells may migrate collectively rather than individually during endometrial fold extension which is a key process leading to uterine lumen closure in pigs.

In the present study, the N‐glycan profiles of pig endometrium during implantation were determined by MALDI‐TOF MS. A total of 36 possible N‐glycan compositions were estimated. The subsequent relative quantification analysis revealed that the sialylated glycans were abundantly expressed. To our knowledge, this is the first report on the identification of the largest number of N‐glycan compositions from endometrium during implantation in mammals using MS method.

The sialylation plays essential roles in various biological process.^37^, ^38^ Although α2,6‐sialylation alteration has been reported to have impact on endometriosis development in humans,^39^, ^40^ little is known about the role of α2,6‐sialylation in implantation. In this study, we found that α2,6‐linked sialic acid was expressed in uterine LE and that the α2,6‐sialylation was mediated by ST6GAL1, a key sialyltransferase catalysing the α2,6‐sialylation on N‐glycans. Notably, α2,6‐linked sialic acid and ST6GAL1 showed a dynamic change ‘weak‐strong‐undetectable’ in pig uterine LE during the dynamic process of uterine lumen ‘open‐close‐reopen’, implying an association between the expression of α2,6‐linked sialic acid and the closure of uterine lumen. As expected, the expression patterns of α2,6‐linked sialic acid and ST6GAL1 in uterine LE were related to that of Rac1(a regulator of collective cell migration) which was found to be highly expressed in uterine LE during the endometrial fold extension. In addition, our in vitro studies revealed that the α2,6‐sialylation regulated by ST6GAL1 could enhance the collective cell migration through Rac1 pathway. Our findings indicate that ST6GAL1‐mediated α2,6‐sialylation participates in the closure of the uterine lumen by promoting collective migration of uterine LE during the endometrial fold extension.

**FIGURE 8:**
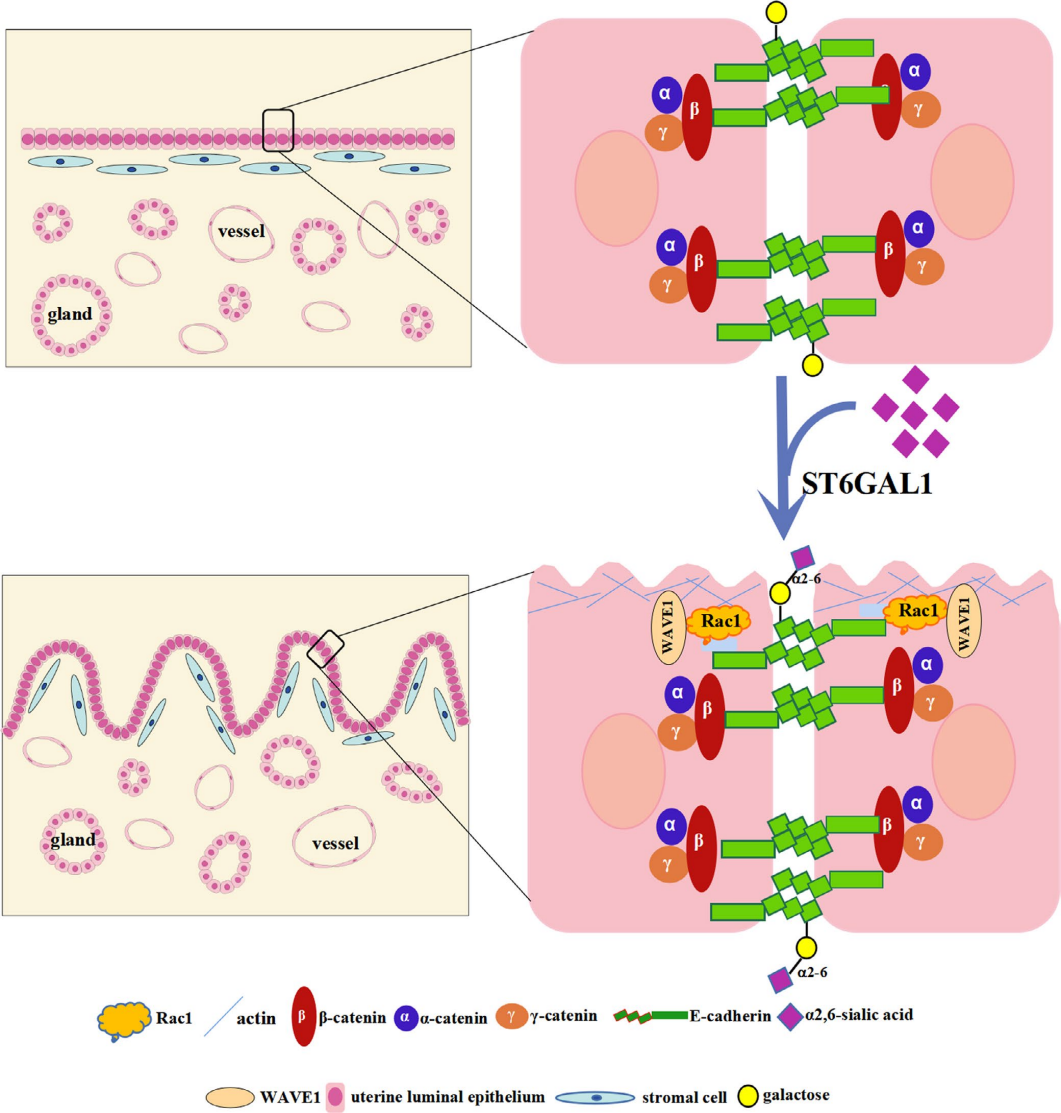
Effect of α2,6‐sialylation mediated by ST6GAL1 on regulation of pig endometrial fold extension during implantation. The increase in α2,6‐sialylation of the cell adhesion molecule E‐cadherin occurs coincident with the activation of Rac1/WAVE1 signal pathway, suggesting that α2,6‐sialylation of E‐cadherin mediated by ST6GAL1 may have a role in regulating endometrial fold extension by promoting the collective migration of uterine LE during implantation

To investigate the mechanisms by which ST6GAL1‐mediated α2,6‐sialylation promoted collective cell migration during implantation in pigs, the α2,6‐sialylated proteins in pig uterine LE were characterized by LC‐MS/MS, and they were found to be mainly involved in cell adhesion, such as E‐cadherin. As a cell‐cell adhesion molecule of epithelial cells, E‐cadherin mediates adhesion junction which plays a vital role in collective cell migration.^33^, ^41^ Previous studies have revealed that the adhesion junction stability is dynamically tuned, allowing the considerable rearrangements of cells during collective cell migration, but the corresponding mechanisms remain to be elucidated.^42^, ^43^ The role of N‐glycosylation in E‐cadherin‐mediated epithelial cell‐to‐cell adhesion, E‐cadherin cellular trafficking and E‐cadherin‐dependent signalling has been confirmed in previous studies.^44^, ^45^ Several reports have demonstrated that the modification of E‐cadherin with high‐mannose, hybrid, sialylated and complex N‐glycans can affect the stability of the adhesion junction, and such an effect is dependent on specific N‐glycan structures linked to E‐cadherin.^46^ However, the sialylation of E‐cadherin was only detected in a canine mammary carcinoma cell line, and its sialylation was positively correlated with malignant phenotype.^47^ In the present study, we demonstrated that E‐cadherin was α2,6‐sialylated in pig uterine LE and Ishikawa cells. In addition, our *in vivo* and *in vitro* data revealed that α2,6‐sialylation level of E‐cadherin was increased with the enhanced collective cell migration mediated by Rac1. Taken together, our results suggest that collective cell migration of pig uterine LE may be modulated by α2,6‐sialylation of E‐cadherin (Figure 8).

## 5 CONCLUSION

Our study indicates that pig uterine luminal epithelial cells may migrate collectively during endometrial fold extension which is a key process leading to uterine lumen closure during implantation. By using MALDI‐TOF MS, we identified a large number of N‐glycan compositions from endometrium during implantation. Furthermore, our study provides evidence to support that ST6GAL1‐mediated α2,6‐sialylation of cell adhesion molecules, such as E‐cadherin, facilitates uterine lumen closure by regulating collective migration of uterine LE during endometrial fold extension. Our finding can provide an insight into embryo implantation in pigs as well as other large animals and humans whose endometrial samples are largely inaccessible during implantation.

## CONFLICT OF INTEREST

The authors have declared that no competing interests exist.

## AUTHOR CONTRIBUTIONS

Mei Yu and Kun Han conceptualized the study. Kun Han, Weijie Dong and Mei Yu involved in formal analysis, methodology and writing—review and editing. Kun Han, Weijie Dong, Feiyu Wang, Yulu Yue, Xihong Tan and Miao Tian involved in investigation. Mei Yu involved in funding acquisition. Mei Yu, Shuhongzhao and Yiliang Miao collected the resources. Kun Han wrote the original draft.

## Supporting information

Fig S1

Fig S2

Fig S3

Fig S4

Fig S5

Table S1

Table S2

Table S3

Supplementary Material

## Data Availability

The mass spectrometry proteomics data have been deposited to the ProteomeXchange Consortium via the PRIDE partner repository with the data set identifier PXD027571. Data are available via ProteomeXchange.
