# Supplementary figures and images for: Glycomics reveal that ST6GAL1‐mediated sialylation regulates uterine lumen closure during implantation
Source: Cell Prolif. 2021 Dec 27;55(1):e13169. doi: 10.1111/cpr.13169 (PMC8780930; doi:10.1111/cpr.13169)

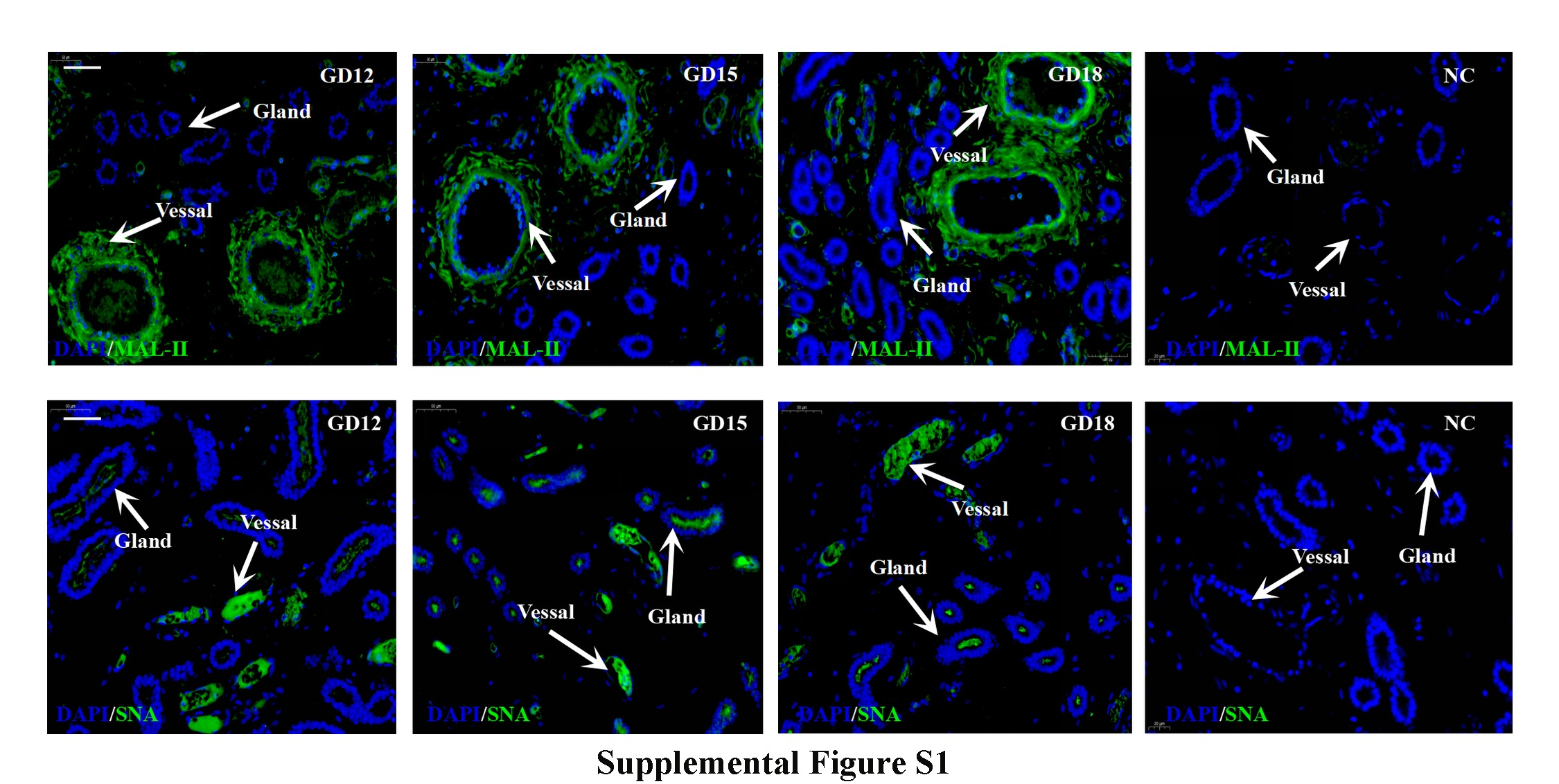

Supplement: Supplementary file 1 — Fig S1 [file CPR-55-e13169-s006.tif]

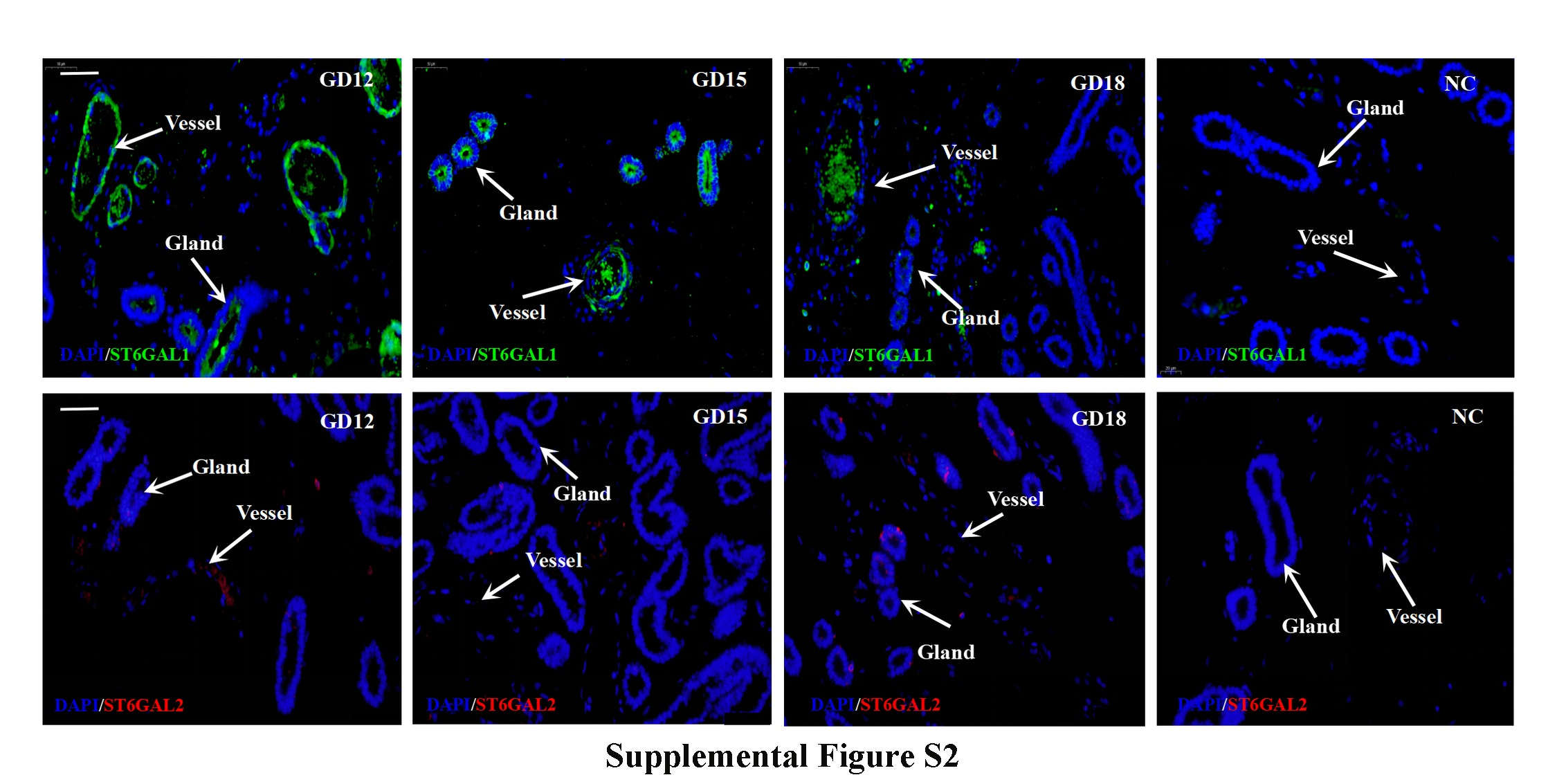

Supplement: Supplementary file 2 — Fig S2 [file CPR-55-e13169-s003.tif]

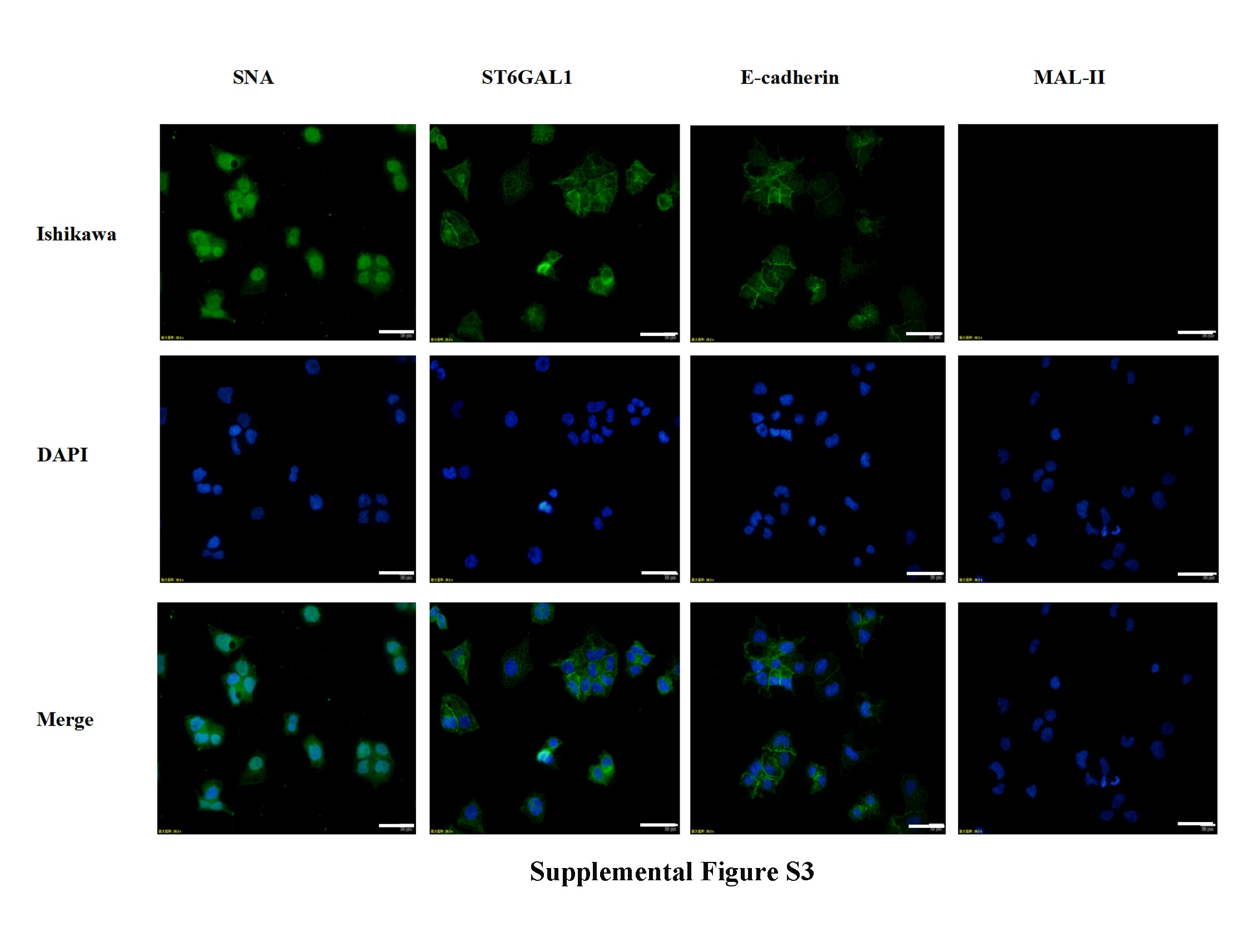

Supplement: Supplementary file 3 — Fig S3 [file CPR-55-e13169-s004.tif]

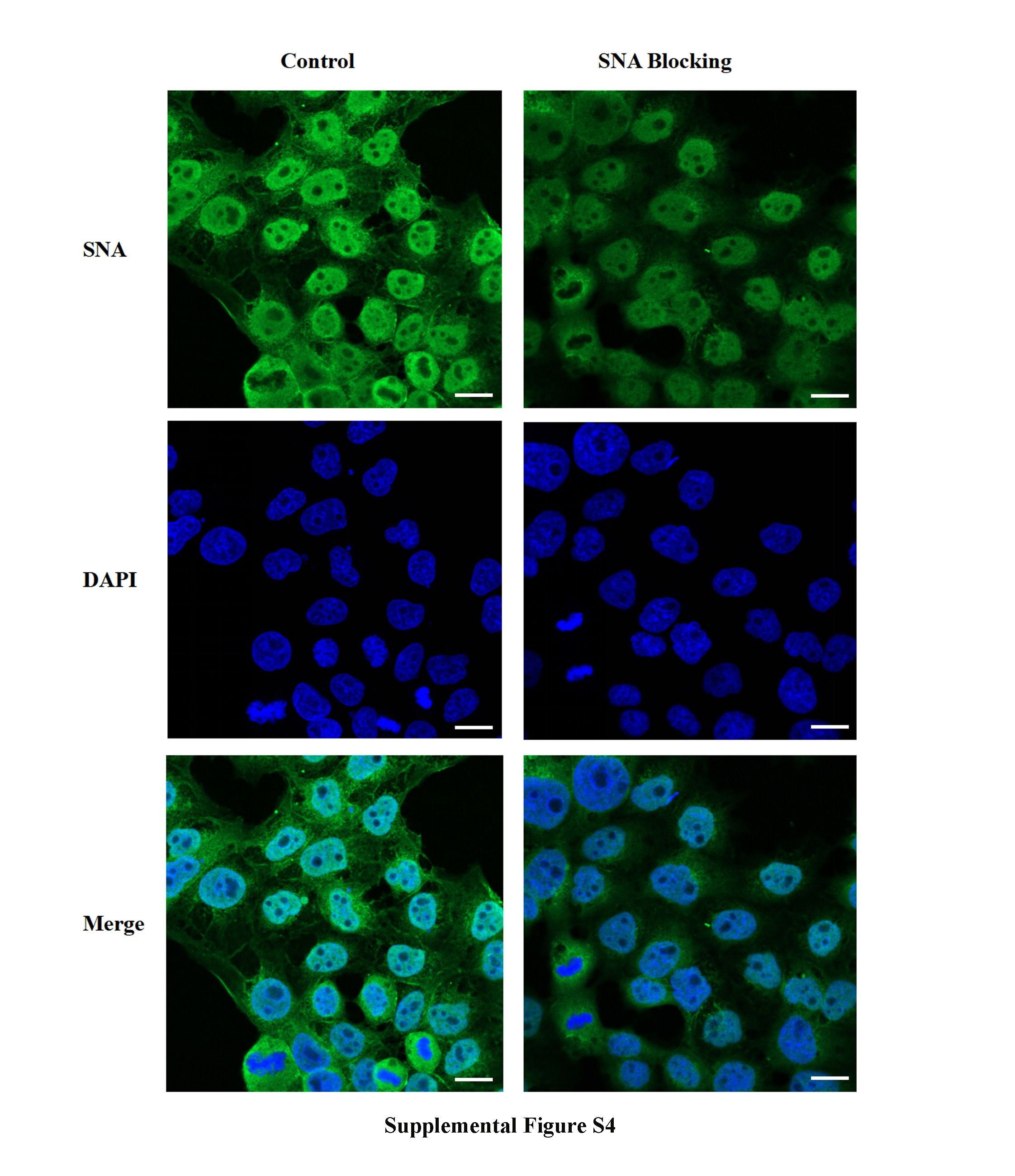

Supplement: Supplementary file 4 — Fig S4 [file CPR-55-e13169-s001.tif]

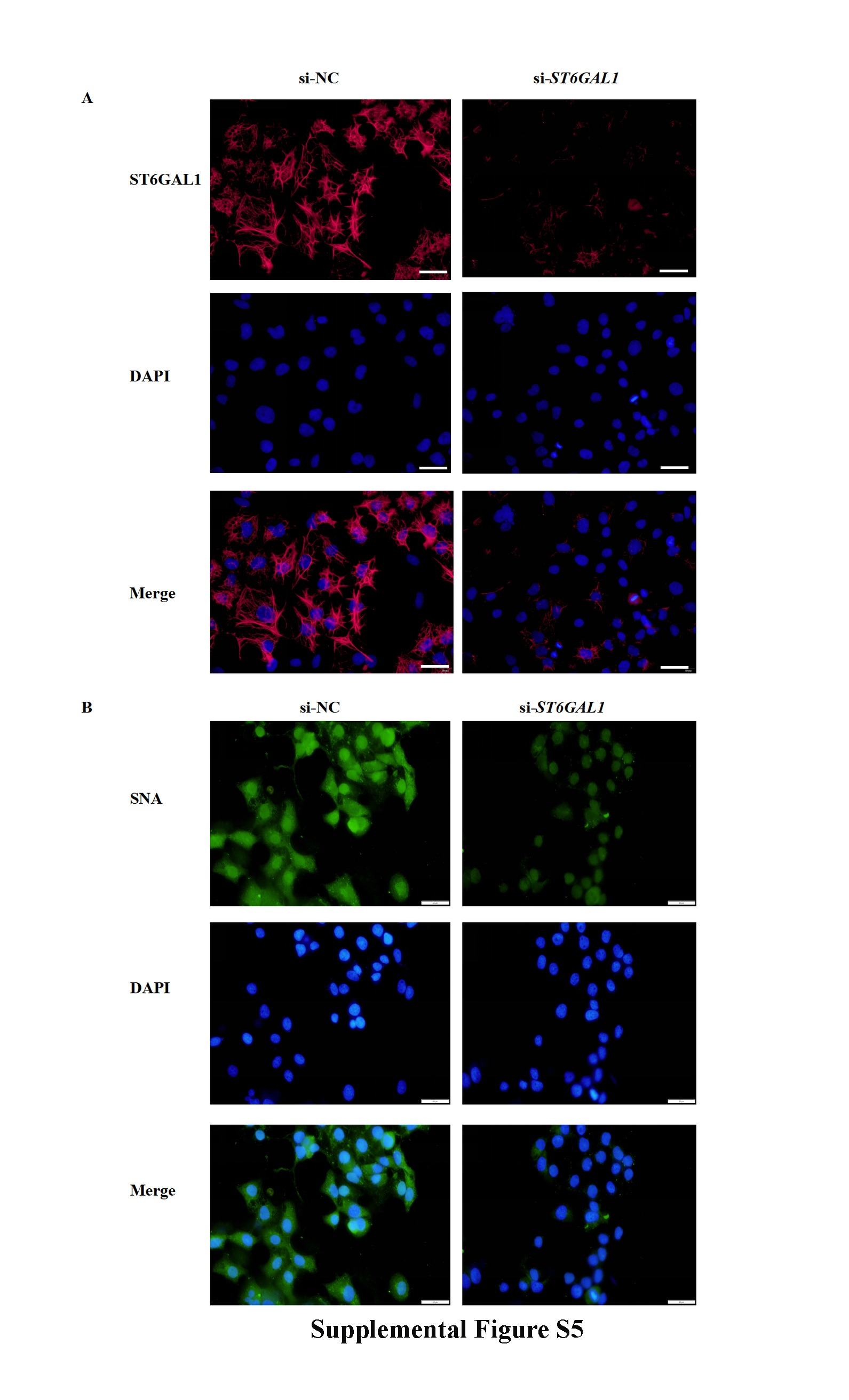

Supplement: Supplementary file 5 — Fig S5 [file CPR-55-e13169-s007.tif]
