# Supplementary material for: Glycomics reveal that ST6GAL1‐mediated sialylation regulates uterine lumen closure during implantation
Source: Cell Prolif. 2021 Dec 27;55(1):e13169. doi: 10.1111/cpr.13169 (PMC8780930; doi:10.1111/cpr.13169)
Supplement: Supplementary file 6 — Table S1 [file CPR-55-e13169-s009.doc]

**Supplemental Table S1. Sequences of qRT-PCR primers used in the study.**

| Gene | Forward Primer (5'-3') | Reverse Primer (5'-3') |
| --- | --- | --- |
| *Sus scrofa-ST6GAL1* | ACAGAACAGCGCTTCCTCAA | CAAGGCATCTGGGGCTTCA |
| *Sus scrofa-ST6GAL2* | CACACGGCTACGAGAAGGAT | TTGCAGAATAAGGAGCTGGGT |
| *Homo sapiens*-*ST6GAL1* | GATTCCCAGTCTGTATCCT | GGTTTTTGGAAGAGCTGT |
