# Supplementary material for: Glycomics reveal that ST6GAL1‐mediated sialylation regulates uterine lumen closure during implantation
Source: Cell Prolif. 2021 Dec 27;55(1):e13169. doi: 10.1111/cpr.13169 (PMC8780930; doi:10.1111/cpr.13169)
Supplement: Supplementary file 7 — Table S2 [file CPR-55-e13169-s002.doc]

**Supplemental Table S2. The relative area of N-glycans released from pig endometriums on gestational days 12, 15 and 18.**

| **Peak** | **Calculated m/z^a)^** | **Relative area** | | | **Estimated monosaccharide composition^b)^** | **Speculative Structure** |
| --- | --- | --- | --- | --- | --- | --- |
|  |  | **GD12** | **GD15** | **GD18** |  |  |
| **1** | **1567.00** | **100.0** | **100.0** | **100.0** | **-** |  |
| **2** | **1649.67** | **57.6** | **80.8** | **29.8** | **(Hex)_2_ + (Man)_3_ (GlcNAc)_2_** |  |
| **3** | **1674.70** | **11.2** | **13.8** | **6.4** | **(HexNAc) (Fuc) + (Man)_3_ (GlcNAc)_2_** |  |
| **4** | **1690.69** | **1.5** | **3.2** | **2.3** | **(Hex) (HexNAc) + (Man)_3_ (GlcNAc)_2_** |  |
| **5** | **1731.72** | **214.0** | **168.2** | **67.2** | **(HexNAc)_2_ + (Man)_3_ (GlcNAc)_2_** |  |
| **6** | **1811.72** | **126.8** | **119.1** | **47.3** | **(Hex)_3_ + (Man)_3_ (GlcNAc)_2_** |  |
| **7** | **1852.75** | **1.9** | **4.6** | **3.2** | **(Hex)_2_ (HexNAc) + (Man)_3_ (GlcNAc)_2_** |  |
| **8** | **1877.78** | **1075.0** | **760.0** | **219.2** | **(HexNAc)_2_ (Fuc) + (Man)_3_ (GlcNAc)_2_** |  |
| **9** | **1893.77** | **67.8** | **108.1** | **36.4** | **(Hex) (HexNAc)_2_ + (Man)_3_ (GlcNAc)_2_** |  |
| **10** | **1973.77** | **68.4** | **57.8** | **22.5** | **(Hex)_4_ + (Man)_3_ (GlcNAc)_2_** |  |
| **11** | **2039.83** | **340.3** | **210.4** | **67.9** | **(Hex) (HexNAc)_2_ (Fuc) + (Man)_3_ (GlcNAc)_2_** |  |
| **12** | **2080.86** | **16.8** | **8.6** | **4.2** | **(HexNAc)_3_ (Fuc) + (Man)_3_ (GlcNAc)_2_** |  |
| **13** | **2135.82** | **57.2** | **39.9** | **16.6** | **(Hex)_5_ + (Man)_3_ (GlcNAc)_2_** |  |
| **14** | **2141.84** | **55.2** | **31.8** | **11.4** | **(Hex) (HexNAc) (Fuc) (Neu5Ac) + (Man)_3_ (GlcNAc)_2_** |  |
| **15** | **2157.83** | **15.5** | **11.0** | **5.7** | **(Hex) (****HexNAc) (Fuc) (Neu5Gc) + (Man)_3_ (GlcNAc)_2_** |  |
| **16** | **2201.88** | **76.8** | **58.9** | **20.2** | **(Hex)_2_ (HexNAc)_2_ (Fuc) + (Man)_3_ (GlcNAc)_2_** |  |
| **17** | **2242.91** | **24.2** | **15.9** | **7.4** | **(Hex) (HexNAc)_3_ (Fuc) + (Man)_3_ (GlcNAc)_2_** |  |
| **18** | **2297.88** | **56.4** | **38.2** | **12.4** | **(Hex)_6_ + (Man)_3_ (GlcNAc)_2_** |  |
| **19** | **2303.89** | **35.2** | **15.2** | **4.4** | **(Hex)_2_ (HexNAc) (Fuc) (Neu5Ac) + (Man)_3_ (GlcNAc)_2_** |  |
| **20** | **2319.89** | **4.3** | **6.5** | **4.4** | **(Hex)_3_ (HexNAc) (Neu5Ac) + (Man)_3_ (GlcNAc)_2_** |  |
| **21** | **2344.92** | **20.1** | **17.1** | **5.8** | **(Hex) (HexNAc)_2_ (Fuc) (Neu5Ac) + (Man)_3_ (GlcNAc)_2_** |  |
| **22** | **2360.91** | **101.0** | **53.6** | **13.8** | **(Hex)_2_ (HexNAc)_2_ (Neu5Ac) + (Man)_3_ (GlcNAc)_2_** |  |
| **23** | **2376.91** | **5.5** | **6.9** | **2.9** | **(Hex)_2_ (HexNAc)_2_ (Neu5Gc) + (Man)_3_ (GlcNAc)_2_** |  |
| **24** | **2404.96** | **21.2** | **14.0** | **5.9** | **(Hex)_2_ (HexNAc)_3_ (Fuc) + (Man)_3_ (GlcNAc)_2_** |  |
| **25** | **2465.94** | **25.8** | **12.7** | **3.3** | **(Hex)_3_ (HexNAc) (Fuc) (Neu5Ac) + (Man)_3_ (GlcNAc)_2_** |  |
| **26** | **2506.97** | **236.5** | **135.7** | **29.5** | **(Hex)_2_ (HexNAc)_2_ (Fuc) (Neu5Ac) + (Man)_3_ (GlcNAc)_2_** |  |
| **27** | **2522.97** | **60.6** | **28.9** | **8.0** | **(Hex)_2_ (HexNAc)_2_ (Fuc) (Neu5Gc) + (Man)_3_ (GlcNAc)_2_** |  |
| **28** | **2548.00** | **24.2** | **11.9** | **3.9** | **(Hex) (HexNAc)_3_ (Fuc) (Neu5Ac) + (Man)_3_ (GlcNAc)_2_** |  |
| **29** | **2666.01** | **185.0** | **100.5** | **12.5** | **(Hex)_2_ (HexNAc)_2_ (Neu5Ac)_2_ + (Man)_3_ (GlcNAc)_2_** |  |
| **30** | **2710.05** | **21.8** | **9.5** | **2.5** | **(Hex)_2_ (HexNAc)_3_ (Fuc) (Neu5Ac) + (Man)_3_ (GlcNAc)_2_** |  |
| **31** | **2726.05** | **3.5** | **4.3** | **1.6** | **(Hex)_3_ (HexNAc)_3_ (Neu5Ac) + (Man)_3_ (GlcNAc)_2_** |  |
| **32** | **2770.09** | **36.8** | **15.7** | **4.9** | **(Hex)_3_ (HexNAc)_4_ (Fuc) + (Man)_3_ (GlcNAc)_2_** |  |
| **33** | **2812.07** | **3618.5** | **2031.0** | **446.2** | **(Hex)_2_ (HexNAc)_2_ (Fuc) (Neu5Ac)_2_ + (Man)_3_ (GlcNAc)_2_** |  |
| **34** | **2828.06** | **183.9** | **318.9** | **57.3** | **(Hex)_2_ (HexNAc)_2_ (Fuc) (Neu5Ac) (Neu5Gc) + (Man)_3_ (GlcNAc)_2_** |  |
| **35** | **2872.10** | **148.3** | **59.2** | **9.3** | **(Hex)_3_ (HexNAc)_3_ (Fuc) (Neu5Ac) + (Man)_3_ (GlcNAc)_2_** |  |
| **36** | **3177.20** | **2.5** | **3.6** | **—** | **(Hex)_3_ (HexNAc)_3_ (Fuc) (Neu5Ac)_2_ + (Man)_3_ (GlcNAc)_2_** |  |
| **37** | **3482.29** | **30.6** | **10.5** | **2.0** | **(Hex)_3_ (HexNAc)_3_ (Fuc) (Neu5Ac)_3_ + (Man)_3_ (GlcNAc)_2_** |  |

Note: “-” indicates not detected. a) The glycans were calculated as the aminooxy tryptophanylarginine labeled derivatives in which neuraminic acids were methylesterified, and [M + H]^+^. b) Monosaccharide compositions were determined by database searching using GlycoWorkbench software. peak 1: an internal standard (maltoheptaose) with a known concentration. Hex, hexose; HexNAc, N-acetyl hexosamine ; Fuc, fucose ; N-acetylglucosamine ; Neu5Ac, N-acetyl neuraminic acid ; Neu5Gc, N-glycolylneuraminic acid ; Man, mannose ; Galactose .
