# Supplementary material for: Glycomics reveal that ST6GAL1‐mediated sialylation regulates uterine lumen closure during implantation
Source: Cell Prolif. 2021 Dec 27;55(1):e13169. doi: 10.1111/cpr.13169 (PMC8780930; doi:10.1111/cpr.13169)
Supplement: Supplementary file 9 — Supplementary Material [file CPR-55-e13169-s005.docx]

**Supplemental information**

**Supplemental Figure S1. Distribution of α2,3-/α2,6-linked sialic acid in stroma, gland and blood vessel in pig endometrium during implantation.** SNA was used to detect α2,6-linked sialic acid; MAL-II was used to detect α2,3-linked sialic acid. The lectin-positive signal is in green, while the nucleus is in blue. NC, negative control. Scale bar = 50 µm.

**Supplemental Figure S2.** **Expression patterns of ST6GAL1 and ST6GAL2 in stroma, gland and blood vessel in pig endometrium during implantation.** NC, negative control. Scale bar = 50 µm.

**Supplemental Figure S3. Expression of α2,3-/α2,6-linked sialic acid, ST6GAL1 and E-cadherin in Ishikawa cells.** SNA was used to detect α2,6-linked sialic acid; MAL-II was used to detect α2,3-linked sialic acid. Scale bar = 50 µm, n=3 independent experiments.

**Supplemental Figure S4. The changes in the expression level of α2,6-linked sialic acid in Ishikawa cells treated with or without SNA.** The lectin-positive signal is in green, while the nucleus is in blue. Scale bar = 50 µm, n=3 independent experiments.

**Supplemental Figure S5. The expression levels of ST6GAL1 (A) and** **α2,6-linked sialic acid (B)** **in Ishikawa cells treated with Si-NC and Si*-ST6GAL1*.** SNA was used to detect α2,6-sialic acid. The positive signal is in green or rose red, while the nucleus is in blue, Scale bar = 50 µm.

**Supplemental Table S1. The sequences of the qRT-PCR primers used in this study.**

**Supplemental Table S2. The composition of N-glycans released from endometrial tissues on gestational days 12, 15 and 18 determined by MALDI-MS analysis.**

**Supplemental Table S3. The α2,6-sialylated proteins identified from pig endometrium on gestational day 15.**
